# Association between the levels of physical activity and plantar pressure in 6-14-year-old children

**DOI:** 10.7717/peerj.8551

**Published:** 2020-02-14

**Authors:** Lovro Štefan, Mario Kasović, Martin Zvonar

**Affiliations:** 1 Department of General and Applied Kinesiology, Faculty of Kinesiology, University of Zagreb, Zagreb, Croatia; 2 Department of Sports Motorics and Methodology in Kinathropology, Faculty of Sports Studies, Masaryk University, Brno, Czech Republic

**Keywords:** Exercise, School students, Force, Contact time, Relationship

## Abstract

**Background:**

The main purpose of the study was to determine whether lower levels of physical activity were associated with higher plantar pressure generated under each foot.

**Methods:**

In this cross-sectional study, we recruited 641 children aged 6–14 years (age_mean ± SD_ = 9.7  ± 2.4 years; height_mean ± SD_ = 143.6  ± 15.3 cm, weight_mean ± SD_ = 37.6  ± 13.4 kg; body-mass index_mean ± SD_ = 17.6  ± 3.2 kg/m^2^; 44.2% girls). We used EMED –XL pressure platform to measure force time integral, pressure-time integral, contact-time and contact area, peak plantar pressure and mean plantar pressure of the right and the left foot during the gait analysis. The level of physical activity was measured by using The Physical Activity Questionnaire for Older Children (PAQ–C). The associations were calculated by using generalized estimating equations with linear regression models.

**Results:**

Lower levels of physical activity were associated with higher force- and pressure-time integrals, longer contact time and higher peak and mean plantar pressures in both feet.

**Conclusion:**

Our study shows that the level of physical activity is strongly and inversely associated with plantar pressure in a sample of 6–14 year olds.

## Introduction

Evidence shows that regular participation in physical activity leads to positive health-related outcomes, such as lower risk of all-cause mortality, cardiovascular, metabolic, musculoskeletal and mental diseases (Warburton, Nicol & Bredin, 2006). The World Health Organization proposes that children aged <18 years should engage in at least 60 min of moderate-to-vigorous intensity physical activity per week (World Health Organization, 2011). Although health benefits of physical activity have been well-documented (Warburton, Nicol & Bredin, 2006), the prevalence of insufficient physical activity in European school-going children is very high and ranges between 80 and 90% (World Health Organization, 2010). Moreover, the prevalence of overweight and obesity in children has significantly increased in the last forty year (NCD-RisC, 2017), and generating higher levels of physical activity may prevent from unhealthy weight gain (Strong et al., 2005).

Despite the encouragement to engage in regular physical activity in children, the structure and function of the foot may be of potential barrier for doing it (Mickle et al., 2011). Specifically, previous studies have shown that overweight and obese individuals carry excessive body mass when participating in physical activity, causing musculoskeletal pain and discomfort in lower extremities (Chan & Chen, 2009). Another study conducted among 3-year-olds has shown that overweight/obese children tend to have flatter feet and generate higher dynamic plantar pressures, compared to non-overweight children (Mickle, Steele & Munro, 2006). Such condition may lead to foot pain and discomfort, and finally to less physical activity. To date, only a small proportion of studies have been examining of how the level of physical activity is associated with foot characteristics in children (Mickle et al., 2011; Riddiford-Harland et al., 2015). Specifically, Mickle et al. (2011) reported a significant inverse association between the peak plantar pressure generated under heel with total physical activity (*r* =  − 0.53) and moderate-to-vigorous physical activity (*r* =  − 0.47) in boys, while in girls, no significant associations were observed. Another study also showed that different intensities of physical activity were inversely associated with middle and lateral forefoot and lateral midfoot pressure in overweight children (Riddiford-Harland et al., 2015). Although both studies objectively measured intensity, frequency and duration of physical activity, they did not capture the level of physical activity done in different time of the day (for example during lunch- or class-breaks, after school and in the evenings), which significantly enhances the recall ability of children (Kowalski, Crocker & Donen, 2004). Also, previous studies have only used peak plantar pressure to assess foot pressure, while other potential determinants, such as force- and pressure-time integrals and contact area and time have not been studied yet. Finally, both studies have been conducted among a small number of participants (*N* = 33 and *N* = 73), which might have underpowered the results in some aspects.

Therefore, the main purpose of the study was to determine whether lower levels of physical activity in different time of the day were associated with plantar pressure determinants generated under each foot in a sample of 6–14 year olds.

## Materials & Methods

### Study participants

In this cross-sectional study, participants were 641 primary-school children (6–14 years (age_mean±SD_ = 9.7 ± 2.4 years; height_mean±SD_ = 143.6 ± 15.3 cm, weight_mean±SD_ = 37.6 ± 13.4 kg; body-mass index _mean±SD_ = 17.6 ± 3.2 kg/m^2^; 44.2% girls) randomly chosen from five public schools in the city of Brno, Czech Republic. At the first stage, we contacted principals from each school to give permission for conducting the study. At the second stage, we introduced children and their parents with measurement protocol, potential contribution of the research, and possible discomforts during the execution of the research. Those children whose parents had given a written informed consent entered the study. All procedures were in accordance with the Declaration of Helsinki. The Faculty of Sports Studies, Masaryk University, Brno, Czech Republic granted ethical approval to carry out the study with in its facilities (Ethical code: 02/2018).

### Physical activity assessment

To assess the level of physical activity, we used Physical Activity Questionnaire for Older Children (PAQ–C). The PAQ–C is a self-administrated, 7–day recall instrument to assess general levels of physical activity throughout the elementary school year (Kowalski, Crocker & Donen, 2004). Children were asked to complete the questionnaire with 10 questions regarding the level of physical activity in: (1) spare time, (2) during physical education, (3) during breaks between classes, (4) during lunch-break, (5) right after school, (6) during evenings, (7) during last weekend, (8) self-evaluated and (9) for each day last week. For question 1, we took the mean of all activity on the activity checklist to form a composite score. From question 2–8, we used the reported value that was checked off for each item (the lowest activity response being a 1 and the highest activity response being a 5). Similarly to question 1, we took the mean of all days of the week to create a composite score for question 9. Finally, when we had a value from 1 to 5 for each of the 9 questions, we took the mean of these 9 questions to create the total physical activity score. A score of 1 indicated low, whereas a score of 5 indicated high level of physical activity (Kowalski, Crocker & Donen, 2004). Kowalski, Crocker & Faulkner (1997a) and Kowalski, Crocker & Kowalski (1997b) showed satisfactory reliability and validity properties of the questionnaire. The reliability of the questionnaire in this study was satisfactory (Cronbach’s *α* = 0.782).

### Plantar pressure distribution

Dynamic plantar distributions generated under left and right foot were quantified as the children walked over a calibrated EMED–XL pressure platform (frequency of 100 Hz, resolution of 4 sensors per cm^2^, 1,440 × 440 mm sensor area and pressure range between 10–1,270 kPa; Novel_gmbh_, Munich, Germany). Children were asked to walk at their normal pace over the platform with previous familiarization, as done in previous studies (Riddiford-Harland et al., 2015). In brief, an adult assisted each participant by holding each participant’s hand as they walked over the platform for the first time, after which the adult walked beside the participants without holding the hand to ensure normal arm swing. Data were collected for three successful trials for each participant’s left and right feet as follows: (i) force-time integral (N*s), (ii) pressure-time integral (kPa*s), (iii) contact area (cm^2^), (iv) contact time (ms), (v) peak pressure (kPa) and (vi) mean pressure (kPa). Of note, the reliability of all three trials for the left and the right foot in all variables (from i to vi) was beyond 0.900.

### Data analysis

Basic descriptive statistics of the study participants are presented as mean (x) and standard deviation (SD). Differences between sexes were examined by using Student *t*-test for independent samples. Since in general no significant differences between sexes in plantar pressure distribution and the level of physical activity were observed, we dropped the sex-stratified analysis. The associations between physical activity and plantar pressure distribution variables were calculated by using generalized estimating equations with linear regression models, robust estimator covariance matrix, exchangeable structure matrix and 100 maximum iterations. The results were presented as beta (*β*) coefficients with 95 percent confidence intervals (95% CI). Two-sided *p*-values were calculated and significance was set at *α* <0.05. All the analyses were performed using Statistical Packages for Social Sciences v.23 (SPSS, Chicago, IL, USA).

## Results

Basic descriptive statistics of the study participants are presented in Table 1. Boys were taller, heavier and had higher body-mass index values, compared with girls (*p* < 0.05). Boys had significantly higher force-time integral and mean plantar pressure values in the left foot and only mean plantar pressure value in the right foot, compared with girls (*p* < 0.05). Of note, we presented the results for both the left and the right foot, since we detected significant differences in force-time integral (*t* =  − 3.86, *df* = 640, *p* < 0.001), contact area (*t* =  − 3.80, *df* = 640, *p* < 0.001), contact time (*t* =  − 2.70, *df* = 640, *p* = 0.007) and mean plantar pressure (*t* = −198, *df* = 640, *p* = 0.048) between the feet. No significant differences in the level of physical activity between sexes were observed (*p* < 0.05), except for the level of physical activity doing in spare time (*p* = 0.007) and during breaks between classes (*p* < 0.001) in favor to boys, compared with girls.

**Table 1:** Basic descriptive statistics of the study participants, Czech Republic.

Study variables	Total (*N* = 641)	Boys (*N* = 357)	Girls (*N* = 284)	*p* –value^*^
	x ± SD	x ± SD	x ± SD	
Age (yrs)	9.7 ± 2.4	9.8 ± 2.4	9.5 ± 2.4	0.160
Height (cm)	143.6 ± 15.3	145.1 ± 15.9	141.7 ± 14.4	0.008
Weight (kg)	37.6 ± 13.4	39.1 ± 14.3	35.6 ± 11.9	0.002
BMI (kg/m^2^)	17.6 ± 3.2	17.9 ± 3.3	17.3 ± 2.9	0.009
Left foot:				
Force-time integral (N^*^ s) (i)	185.3 ± 81.6	191.2 ± 84.6	177.8 ± 77.0	0.040
Pressure-time integral (kPa^*^s) (ii)	132.9 ± 43.0	135.4 ± 43.4	129.8 ± 42.5	0.103
Contact area (cm^2^) (iii)	108.7 ± 21.8	109.8 ± 22.2	107.4 ± 21.3	0.166
Contact time (ms) (iv)	634.1 ± 86.1	637.2 ± 85.6	630.3 ± 86.6	0.313
Peak pressure (kPa) (v)	408.3 ± 130.1	415.2 ± 133.1	400.0 ± 126.1	0.134
Mean pressure (kPa) (vi)	83.1 ± 13.4	84.2 ± 14.2	81.8 ± 12.2	0.023
Right foot:				
Force-time integral (N*s) (i)	186.8 ± 81.9	192.1 ± 83.6	180.1 ± 70.3	0.066
Pressure-time integral (kPa^*^s) (ii)	132.6 ± 44.1	134.1 ± 43.2	131.1 ± 45.2	0.386
Contact area (cm ^2^) (iii)	109.6 ± 21.8	110.7 ± 21.6	108.3 ± 21.9	0.159
Contact time (ms) (iv)	637.0 ± 87.1	638.9 ± 86.7	634.7 ± 87.6	0.539
Peak pressure (kPa) (v)	406.2 ± 129.6	409.4 ± 127.9	402.7 ± 132.8	0.476
Mean pressure (kPa) (vi)	84.0 ± 16.6	85.2 ± 19.1	82.4 ± 12.5	0.030
Physical activity:				
In spare time (1)	1.4 ± 0.3	1.5 ± 0.4	1.4 ± 0.2	0.024
During physical education (2)	4.3 ± 0.9	4.3 ± 0.9	4.3 ± 1.0	0.410
During breaks between classes (3)	2.3 ± 1.2	2.5 ± 1.2	2.1 ± 1.1	0.001
During lunch-break (4)	2.3 ± 1.2	2.4 ± 1.2	2.2 ± 1.2	0.082
Right after school (5)	2.9 ± 1.3	2.9 ± 1.3	2.9 ± 1.3	0.935
During evenings (6)	2.8 ± 1.2	2.8 ± 1.2	2.7 ± 1.1	0.306
During last weekend (7)	2.7 ± 1.0	2.7 ± 1.1	2.7 ± 1.0	0.483
Self-evaluated (8)	2.9 ± 1.2	2.9 ± 1.2	2.8 ± 1.1	0.773
For each day last week (9)	2.8 ± 1.0	2.8 ± 1.0	2.7 ± 1.0	0.582
Total level (10)	2.7 ± 0.6	2.8 ± 0.7	2.7 ± 0.6	0.098

**Notes.**

* Differences were calculated by using Student *t*-test for independent (sex) samples *p* < 0.05.

Tables 2 and 3 present the associations between all physical activity components and plantar pressure distribution variables in the left and the right foot. For the left foot, higher physical activity done in spare time was significantly associated with lower contact time. Also, higher level of physical activity during lunch break was significantly associated with lower force-time integral and contact time, while higher physical activity level after school was significantly associated with lower force-time integral, pressure-time integral, contact time, peak pressure and mean pressure. Moreover, higher level of physical activity during evenings was significantly associated with lower force-time integral, pressure-time integral, contact time and mean pressure. Higher levels of physical activity during last weekend were significantly associated with lower force-time integral and mean pressure, while higher self-evaluated level of physical activity was significantly associated with lower pressure-time integral, peak pressure and mean pressure. Higher physical activity levels done for each day last week were significantly associated with lower force-time integral and pressure-time integral, peak pressure and mean pressure. Finally, higher levels of total physical activity were significantly associated with lower force-time integral, pressure-time integral and mean pressure. Similar associations between the level of physical activity and plantar pressure variables were observed for the right foot.

**Table 2:** Associations between physical activity and the plantar pressure components of the left foot, Czech Republic.

Study variables	Force-time integral	Pressure-time integral	Contact area	Contact time	Peak pressure	Mean pressure
Physical activity:	*β* (95% CI)	*β* (95% CI)	*β* (95% CI)	*β* (95% CI)	*β* (95% CI)	*β* (95% CI)
In spare time	−7.2 (−18.8 to 4.4)	−4.0 (−10.0 to 2.0)	1.0 (−2.5 to 4.5)	−11.6 (−19.2 to −3.9)^**^	−0.6 (−8.7 to 7.4)	0.5 (−2.2 to 3.2)
During physical education	−0.5 (−10.0 to 9.0)	0.6 (−2.4 to 3.6)	0.4 (−1.3 to 2.2)	0.3 (−18.6 to 19.1)	0.4 (−11.3 to 12.1)	−0.2 (−0.5 to 0.1)
During breaks between classes	1.3 (−4.3 to 6.9)	0.7 (−2.7 to 4.2)	0.8 (−1.0 to 1.3)	0.6 (0.1 to 1.2)	3.0 (−12.2 to 18.2)	−0.1 (−1.2 to 1.1)
During lunch-break	−1.8 (−3.0 to −0.7)^**^	−1.6 (−4.3 to 1.1)	0.5 (0.1 to 0.9)^*^	−3.7 (−5.4 to −1.9)^***^	−3.1 (−17.2 to 11.1)	0.1 (−1.0 to 1.3)
Right after school	−5.3 (−7.9 to −2.7)^***^	−3.0 (−5.9 to −0.1)^*^	−0.1 (−0.7 to 0.6)	−2.5 (−4.7 to −0.4)^*^	−6.9 (−12.7 to −1.2)^*^	−0.9 (−0.9 to −0.9)^***^
During evenings	−3.6 (−4.4 to −2.7)^***^	−2.9 (−5.1 to −0.8)^**^	0.3 (−0.4 to 0.5)	−1.8 (−2.4 to −1.2)^***^	−7.1 (−15.5 to 1.3)	−0.7 (−0.8 to −0.6)^***^
During last weekend	−5.9 (−10.4 to −1.4)^**^	−1.5 (−4.9 to 0.7)	−0.5 (−1.4 to 0.4)	−0.4 (−9.3 to 8.5)	−2.1 (−6.6 to 2.3)	−1.2 (−1.3 to −1.1)^***^
Self-evaluated	−1.5 (−6.0 to 3.0)	−3.4 (−3.9 to −3.0)^***^	1.1 (−0.9 to 3.1)	−4.4 (−9.5 to 0.7)	−7.8 (−16.3 to −0.1)^*^	−0.8 (−1.0 to −0.6)^***^
For each day last week	−6.0 (−11.6 to −0.5)^*^	−4.2 (−7.6 to −0.7)^*^	−0.6 (−2.3 to 1.1)	−4.3 (−14.2 to 5.5)	−12.2 (−16.2 to −8.2)^***^	−1.1 (−1.4 to −1.3)^***^
Total level	−8.0 (−13.8 to −2.2)^***^	−5.4 (−6.7 to −4.2)^***^	0.4 (−1.7 to 2.5)	−6.0 (−20.1 to 8.1)	−12.2 (−28.5 to 4.1)	−1.6 (−2.4 to −0.8)^***^

**Notes.**

*β*; unstandardized beta coefficient.

95% CI; 95 percent confidence interval.

*** *p* < 0.001.

** *p* < 0.01.

* *p* < 0.05.

**Table 3:** Associations between physical activity and the plantar pressure components of the right foot, Czech Republic.

Study variables	Force-time integral	Pressure-time integral	Contact area	Contact time	Peak pressure	Mean pressure
Physical activity:	*β* (95% CI)	*β* (95% CI)	*β* (95% CI)	*β* (95% CI)	*β* (95% CI)	*β* (95% CI)
In spare time	−7.5 (−19.7 to 4.7)	−8.1 (−14.7 to −1.5)^*^	2.1 (−2.2 to 6.5)	−12.2 (−17.6 to −6.8)^***^	−25.8 (−33.4 to −18.2)^***^	−1.6 (−4.0 to 0.9)
During physical education	−1.3 (−10.8 to 8.2)	0.5 (−3.7 to 4.7)	0.3 (−1.2 to 1.9)	−0.2 (−19.9 to 19.4)	0.4 (−5.5 to 6.4)	0.00 (−0.17 to 0.17)
During breaks between classes	1.2 (−3.7 to 6.2)	0.1 (−1.2 to 1.4)	0.1 (−1.2 to 1.4)	−0.1 (−2.4 to 2.1)	0.5 (−6.4 to 7.5)	−0.4 (−0.4 to −0.4)^***^
During lunch-break	−1.8 (−2.5 to −1.2)^***^	0.4 (−1.9 to 2.6)	0.7 (0.5 to 0.9)^***^	−3.8 (−3.9 to −3.6)^***^	2.5 (−7.0 to 12.0)	−0.7 (−0.8 to −0.5)^***^
Right after school	−5.5 (−7.9 to −3.0)^***^	−3.5 (−5.6 to −1.5)^***^	0.1 (−0.6 to 0.8)	−3.0 (−4.6 to −1.5)^***^	−7.1 (−7.2 to 7.0)^***^	−1.8 (−2.6 to −1.0)^***^
During evenings	−3.5 (−4.6 to −2.5)^***^	−3.1 (−4.4 to −1.8)^***^	0.3 (−0.1 to 0.8)	−1.7 (−2.1 to −1.2)^***^	−8.8 (−16.6 to −1.0)^*^	−1.9 (−2.8 to −1.0)^***^
During last weekend	−5.5 (−10.0 to −1.0)^*^	−2.7 (−6.6 to 1.2)	−0.3 (−1.1 to 0.5)	0.6 (−6.9 to 8.2)	−3.5 (−7.3 to 0.4)	−2.2 (−3.2 to −1.2)^***^
Self-evaluated	−2.1 (−5.5 to 1.2)	−3.4 (−3.9 to −2.9)^***^	1.1 (−0.1 to 2.4)	−4.8 (−7.7 to 2.0)^***^	−6.9 (−11.7 to 2.0)^**^	−1.2 (−1.7 to −0.6)^***^
For each day last week	−6.5 (−10.5 to −2.5)^***^	−3.5 (−6.8 to −0.2)^*^	−0.6 (−1.4 to 0.2)	−5.1 (−11.7 to 1.6)	−5.3 (−8.5 to −2.1)^***^	−1.4 (−1.8 to −1.0)^***^
Total level	−8.5 (−13.9 to −3.1)^***^	−5.6 (−7.9 to −3.2)^***^	0.8 (−0.6 to 2.2)	−6.7 (−18.7 to 5.2)	−10.7 (−22.6 to −0.1)^*^	−2.6 (−3.6 to −1.5)^***^

**Notes.**

*β*; unstandardized beta coefficient.

95% CI; 95 percent confidence interval.

*** *p* < 0.001.

** *p* < 0.01.

* *p* < 0.05.

## Discussion

The main purpose of the study was to determine whether lower levels of physical activity in different time of the day were associated with plantar pressure determinants generated under each foot in a sample of 6-14 year olds.

Our findings of the significant negative association between the level of physical activity and plantar pressure are in line with previous studies (Mickle et al., 2011; Riddiford-Harland et al., 2015), although we did not collect the data regarding region–specific foot area, or observe different intensities of physical activity, like done in studies described below. Specifically, a study by Mickle et al. (2011) conducted in preschool children showed, that the total physical activity and moderate–to–vigorous physical activity were inversely correlated with the peak plantar pressures under the heel in boys (*r* =  − 0.53 and *r* =  − 0.47), yet no significant correlations were observed in girls. Also, the same group of authors showed a significant positive correlation between the time spent in sedentary behaviour and peak plantar pressures under the 2–5 toe (*r* = 0.53) only in girls. Another study conducted among 73 overweight/obese children aged 8.3 years showed that moderate, vigorous and moderate–to–vigorous physical activity were inversely correlated with higher peak plantar pressures, especially observed under the forefoot area (Riddiford-Harland et al., 2015). Although we did not collect additional data regarding foot pain, previous studies have speculated that children who experience higher plantar pressure values beneath each region of the foot may suffer from more pain during vigorous–intensity type of activities (weight-bearing), which may also be a potential barrier for regular physical activity engagement (Mickle et al., 2011). Similar associations have been confirmed previously in adult population (Hills et al., 2001; Hodge, Bach & Carter, 2005; Hitt et al., 2007). For example, Hitt et al. (2007) showed that individuals with higher plantar pressure participated in lower levels of physical activity as a result of pain and discomfort, especially in their lower limbs. On the other hand, studies have also shown that weight reduction leads to less pain and discomfort and higher participation in physical activity (Hodge, Bach & Carter, 2005). It that way, weight-management policies and strategies which aim to control weight status, especially in children and adolescents, should be implemented within the school system.

This study has several strengths. First, we included a relatively large number of 6-14-year-old children from five randomly selected primary schools. Second, we used objective methods to assess the level of plantar pressure distribution in both feet. In addition, we presented results regarding force–and pressure-time integrals and contact time and area, while previous similar studies have only used peak pressure values (Mickle et al., 2011; Riddiford-Harland et al., 2015). Third, although we used a subjective method to assess the level of physical activity, we captured different times of the day when physical activity was done, which strengthen the overall physical activity level score.

However, this study has a few limitations. First, due to a cross–sectional design of the study, the causality of the association cannot be established, that is, higher plantar pressure levels led to lower levels of physical activity. In addition, previous studies have shown that pain often mediates the association between physical activity and plantar pressure (Hodge, Bach & Carter, 2005; Hitt et al., 2007). Considering our study, we can only speculate that pain and discomfort of the foot discouraged children to participate in higher levels of physical activity. Third, previous studies categorized the foot according to different regions (Mickle et al., 2011; Riddiford-Harland et al., 2015), while we presented the results capturing the foot as a unit. Fourth, although we stated the strength of the PAQ—C, Kowalski, Crocker & Donen (2004) highlighted that ‘the PAQ—C does not provide an estimate of caloric expenditure or specific frequency, time, and intensity information and cannot discriminate between specific activity intensities, such as moderate or vigorous activities’. Finally, we did not collect biomechanical characteristics of the foot, nor gait velocity (Segal et al., 2004; Taylor, Menz & Keenan, 2004; Burnfield et al., 2004; Rosenbaum et al., 1994). Specifically, the aforementioned studies have shown that faster walking speed increases the level of peak and mean plantar pressures in both younger (Segal et al., 2004; Taylor, Menz & Keenan, 2004; Rosenbaum et al., 1994) and older adults (Burnfield et al., 2004) in almost all regions beneath the foot. As for younger children, a recent systematic review and meta-analysis has shown that they walk slower, compared to older ones, which lowers the cadence, step and stride length “and decreases vertical, baking and propulsive forces” (Fukuchi, Fukuchi & Duarte, 2019). On the other hand, younger children have smaller feet. Therefore, these two variables tend to counteract each other in terms of their effect on plantar pressure; i.e., although younger children walk slower, they need to distribute forces under smaller foot area and may potentially generate higher pressures beneath different foot regions. However, previous studies using similar methodology have shown that peak and mean plantar pressures under the foot region appear to be less influenced by gait velocity (Taylor, Menz & Keenan, 2004). Despite that, the same group of authors have suggested that gait velocity needs to be considered when interpreting plantar pressure recordings for variables strongly associated with contact durations, like force-time integrals and pressure-time integrals. Future studies should: (1) determine a significant and clinically relevant plantar pressure threshold which limits the level of physical activity through reference-based values and normative charts and (2) use objective measures to assess both plantar pressure and physical activity level (including questionnaires) over a longer period of time, to determine both genetic and environmental factors which may influence plantar pressure and physical activity association.

## Conclusions

Our study shows that the higher levels of physical activity done in different time of the day and total physical activity levels are significantly associated with lower plantar pressure variables, including peak and mean plantar pressure in a sample of 6–14 year olds. Of all physical activity variables, total level of physical activity was the strongest predictor for several plantar pressure distribution variables, followed by the level of physical activity done in spare time. Although we cannot conclude the causality of the association, special interventions aiming to reduce plantar pressures or to increase the level of organized physical activity should be implemented within the school system.

##  Supplemental Information

10.7717/peerj.8551/supp-1 Supplemental Information 1 Raw data

## Additional Information and Declarations

### Data Availability

The following information was supplied regarding data availability: Anonymized raw data is available in a Supplementary Files.
